# Recent Progress of Mycotoxin in Various Food Products—Human Exposure and Health Risk Assessment

**DOI:** 10.3390/foods14050865

**Published:** 2025-03-03

**Authors:** Kailin Li, Hua Cai, Baozhang Luo, Shenggang Duan, Jingjin Yang, Nan Zhang, Yi He, Aibo Wu, Hong Liu

**Affiliations:** 1 Shanghai Municipal Center for Disease Control and Prevention, Shanghai 200336, China; kailinli2023@163.com (K.L.); caihua@scdc.sh.cn (H.C.); luobaozhang@scdc.sh.cn (B.L.); duanshenggang@scdc.sh.cn (S.D.); yangjingjin@scdc.sh.cn (J.Y.); zhangnan@scdc.sh.cn (N.Z.); heyi@scdc.sh.cn (Y.H.); 2 SIBS-UGENT-SJTU Joint Laboratory of Mycotoxin Research, CAS Key Laboratory of Nutrition, Metabolism and Food Safety, Shanghai Institute of Nutrition and Health, University of Chinese Academy of Sciences, Chinese Academy of Sciences, Shanghai 200331, China

**Keywords:** contaminants, human biomonitoring, population subgroups, health effects

## Abstract

Mycotoxins, as prevalent contaminants in the food chain, exhibit diverse toxicological effects on both animals and humans. Chronic dietary exposure to mycotoxin-contaminated foods may result in the bioaccumulation of these toxins, posing substantial public health risks. This review systematically examines the contamination patterns of mycotoxins across major food categories, including cereals and related products, animal-derived foods, fruits, and medical food materials. Furthermore, we critically evaluated two methodological frameworks for assessing mycotoxin exposure risks: (1) dietary exposure models integrating contamination levels and consumption data and (2) human biomonitoring approaches quantifying mycotoxin biomarkers in biological samples. A key contribution lies in the stratified analysis of exposure disparities among population subgroups (adults, teenagers, children, and infants). Additionally, we summarize current research on the relationship between human mycotoxin biomonitoring and associated health impacts, with a particular emphasis on vulnerable groups such as pregnant women and infants. By elucidating the challenges inherent in existing studies, this synthesis provides a roadmap for advancing risk characterization and evidence-based food safety interventions.

## 1. Introduction

Mycotoxins are fungal secondary metabolites produced during fungal infection of plant hosts, exhibiting toxicity in plants, animals, and humans. Owing to their stable physical and chemical properties, mycotoxins are difficult to eliminate during the traditional processing of cereals, fruits, and vegetables [1] and are subsequently ingested by the human body through dietary intake. In addition, after animals ingest feed containing mycotoxins, toxin residues in animal products such as milk [2] and eggs [3] will be ingested and pose health threats to humans. The spectrum of foodborne mycotoxins encompasses trichothecenes (e.g., deoxynivalenol (DON), T-2 toxins), aflatoxins (AFs) (AFB1, AFB2, AFG1, and AFG2), ochratoxins (OTs) (OTA, OTB), zearalenone (ZEN), fumonisins (FBs) (FB1, FB2, and FB3), patulin (PAT), citrinin (CIT), and other emerging toxins such as *Alternaria* toxins (ATs) (alternariol (AOH), alternariol monomethyl ether (AME), tenuazonic acid (TeA), tentoxin (TEN), and altenuene (ALT)), enniatins (ENNs) (ENNB, ENNB1, ENNA, and ENNA1), and beauvericin (BEA). These compounds demonstrate species-specific toxicological profiles in animals and humans.

Deoxynivalenol (DON) exhibits acute toxicity to both humans and animals and induces stomach upset, vomiting, diarrhea, refusal to feed, dizziness, headache, and nervous disorder, as well as animal abortion and stillbirth. Additionally, DON can damage the immune and reproductive functions of humans and animals, and may even cause the death of animals [4,5]. Aflatoxins (AFs) are hepatotoxic and immunotoxic. They are classified as a Group I carcinogen by the International Agency for Research on Cancer (IARC) [6] and cause stunting and pose other potential threats to the health of humans and animals [7]. OTA has a detrimental impact on human physiological health, including hepatotoxicity, nephrotoxicity, neurotoxicity, and immunotoxicity. It is listed as a Class II carcinogen by the IARC [8]. ZEN induces premature puberty, infertility, abortion, and other phenomena, resulting in reproductive toxicity [9,10]. High-dose exposure further induces damage to multiple organs [9]. Fumonisins (FBs) have potential chronic toxicity, including hepatorenal toxicity, immunosuppressive effects, and neurodevelopmental toxicity [11,12]. Patulin (PAT) shows toxicity to human organs such as the liver, kidneys, and intestines [13] and is classified by the IARC as a Group III carcinogen [14]. *Alternaria* toxins (ATs), which have been less studied, also have non-negligible cytotoxicity and genotoxicity on human gastrointestinal cells [15]. ENNs and BEA have also been demonstrated to be cytotoxic to human cells, including gastric (NCI-N87), intestinal (Caco-2), hepatic (Hep-G2), and renal (Hek-293) cells [16].

Although the level of mycotoxin contamination varies considerably between regions, the types of contaminants can be classified according to food categories. In cereals and cereal products, maize is mainly contaminated with AFBs, FB1, FB2, FB3, T-2, HT-2, NIV, DON, ZENs, ATs, CIT, and BEA; rice is mainly contaminated with ZEN; wheat is mainly contaminated with T-2, HT-2, DON, NIV, ZEN, CIT, FB2, OTA, ATs, ENNs, and BEA [17]; barley is mainly contaminated with DON, NIV, ZEN, HT-2, AFG2, T-2, and OTA; oats are mainly contaminated with DONs, NIV, ZEN, T-2; rye is mainly contaminated with DON, NIV, HT-2; millet is mainly contaminated with FB1, FB2, DONs, ZENs, HT-2, and ATs; sorghum is mainly contaminated with FBs, DONs, and ZENs. Of the global crop supply, an estimated 60–80% of crops are contaminated with mycotoxins, with 20% exceeding the European Union (EU) legal food safety limits [18].

For animal products, AFM1 (the main metabolite of AFB1) is legally monitored in milk by some countries due to its higher levels, and many studies have reported that AFs are heat-stable mycotoxins that can persist during the processing of milk and related dairy products, such as cheese. Also, OTA, FBs, ZEN, DON, and their metabolites have been found in milk and cheese samples in many countries. The most commonly reported mycotoxins in fresh meat and animal organs are AFs, OTA, FBs, and ZEN, whereas the most frequently detected mycotoxins in cured and fermented meat products are OTA and AFs, mainly AFB1. Aquatic products such as fish and derived products are mainly contaminated with AFB1, OTA, DON, FBs, and ZEN [19].

Fresh fruits and vegetables are mainly contaminated with ATs and FBs [20,21]. In particular, thin-skinned and watery fruits such as sweet cherries and tomatoes are more susceptible to contamination by various ATs [22]. Among dried fruits and jams, AFs, OTA, and T-2 are major food contaminants [23,24,25]. Data from China showed that AFs, especially AFB1, were the most frequently detected among different spice samples [26]. In addition, data from Russia indicate a high occurrence of AFs, TEN, OTA, and FB2 in spices [27].

To date, epidemiological studies of mycotoxin exposure have been conducted on household pets, domestic livestock, mice, and human populations [28,29,30]. AFs were first reported and were mainly related to carcinoma [28]. Subsequently, risk assessment reports discussed the occurrence of ZEN, OTA, AFs, and FBs [30,31,32,33,34,35,36]. The specific objectives of the present review are to (i) systematically evaluate dietary-exposure-based risk assessment of mycotoxins, (ii) synthesize biomarker-driven studies utilizing human biological samples (e.g., serum, urine, breast milk) to quantify internal exposure doses, and (iii) critically appraise epidemiological evidence linking mycotoxin exposure to adverse health effects and discuss the challenges in current mycotoxin risk assessment frameworks.

## 2. Risk Assessment of Mycotoxin Exposure by Food Contamination and Consumption Data

Dietary intake is the main route of mycotoxin exposure, and dietary exposure assessment for mycotoxins is generally based on the level of mycotoxin contamination of commercially available food products and the consumption of that food category. Although dietary exposure to mycotoxins is common, the exposure level of mycotoxins is not high enough to exceed internationally recognized thresholds of concern. This review focuses on summarizing studies in which published data on mycotoxin exposure levels posed health risks.

Estimated daily intake (EDI) or probable daily intake (PDI) is usually used to indicate risk characterization [37,38]. In general, dietary intake was compared with the provisional maximum tolerable daily intake (PMTDI), tolerable daily intake (TDI), or tolerable weekly intake (TWI) set by the Joint FAO/WHO Expert Committee on Food Additives (JECFA), or the threshold of toxicological concern (TTC) set by the European Food Safety Authority (EFSA) to estimate health risks [39] (Table 1). The hazard quotient (HQ), based on the ratio of EDI/TDI, and the margin of exposure (MOE), based on the ratio of a toxicological threshold from animals (e.g., benchmark dose limit) and the estimated human exposure, are the two most commonly used ways for characterizing non-carcinogenic, carcinogenic, and genotoxic risk. HQ < 1 or MOE < 10,000 is considered to indicate no risk. Smaller values of MOE mean higher risks. For carcinogenic AFs, there is no acceptable daily intake (ADI) because of their genotoxicity and carcinogenicity. Their risk assessment is performed based on dietary exposure and their primary liver-cancer-causing potency [40]. Notably, JECFA established an acceptable value of 1 ng/kg·bw/day for AFM1—the metabolite of AFB1—to mitigate liver cancer risk [41]. Given the toxicity of mycotoxins, a health risk assessment of human exposure via dietary intake is necessary for food quality and safety supervision and control.

**Figure 1:**
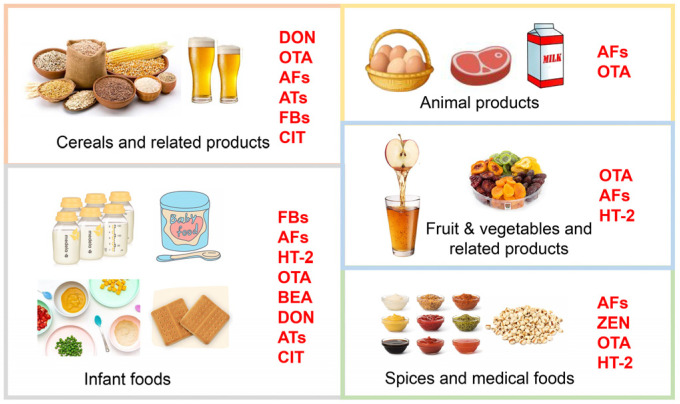
Mycotoxins for which dietary intake exceeds the threshold of concern in different food groups.

Mycotoxin exposure in finished food products covers almost all food categories, among which cereals and related products, animal products, fruits and related products, tea, spices, and even infant foods are of concern as their contamination with certain mycotoxins has exceeded the health reference values (Figure 1) (Table 2, Table 3 and Table 4).

### 2.1. Cereals and Related Products

Mycotoxin contamination in cereals mainly originates from the pre-harvest period, and the accumulation level of toxins is generally higher in the chaff portion [68]. Common post-harvest removal and cleaning can remove up to 50% of the mycotoxins, but inappropriate storage conditions [69,70] and processing are likely to lead to increased contamination levels. For unprocessed and processed cereals, mycotoxin limits were proposed by the European Union and many countries, which largely decrease mycotoxin exposure via the intake of cereals and related products. However, there may be health risks for people with a high intake of cereal products and highly sensitive people. Data on cereal products in Brazil reported that chronic exposure exceeded the PMTDI value at the 95th percentile (P95) for DON and P99 for FBs in the general population, whereas the safe level exceedance occurred at P97.5 for FBs and at P95 for DON for teenagers, as well as at the P99 for FBs for women of child-bearing age [45].

Wheat is one of the main dietary objects among cereals exposed to mycotoxins. Excessive mycotoxin exposure via wheat consumption has been reported mainly in developing countries, including China and Brazil [46], and Portugal and Poland (Table 2). Studies from China demonstrated that the number of cases of adverse health effects (vomiting or diarrhea) due to DON exposure in wheat from some areas exceeded 0.1% [47], and wheat contributed up to 86% of the total DON exposure to humans [45,71]. ATs were also the main contaminants in wheat and related products. The estimated dietary exposure to AME and AOH in wheat grains ranged from 0.003 to 0.007 µg/kg·bw/day, exceeding the TTC value of 0.0025 µg/kg·bw/day, demonstrating potential dietary risks for Chinese consumers [48]. ALT was also reported to pose health risks based on food contamination and consumption data of Shanghai residents [72]. In addition, the calculated estimated weekly intake (EWI) of OTA in beers, in the worst-case scenario, based on a high concentration, exceeded the standard value by 38% [49].

Excessive mycotoxin exposure via maize consumption has also been reported mainly in developing countries including Mexico and African countries (Table 2). Data from Mexico City revealed that the recommended value of mycotoxins was exceeded to a greater extent in the male population due to a higher consumption of maize. Regarding FB1 + FB2, nearly 50% of men and 30% of women might exceed the PMTDI of 2 µg/kg·bw/day for FBs, and for DON, 9% of men and 5% of women would exceed the PMTDI of 1 µg/kg·bw/day [50]. Maize and related products account for up to 91% of the total human exposure to FBs [45]. Studies from North-Central Nigeria reported that chronic exposure to AFs and CIT in maize may result in as many as 33 new liver cancer cases per year per 100,000 population [51].

For rice, data from Belgium demonstrated the potential health risks of CIT and OTA through individual and combined dietary exposure, particularly in relation to rice consumption [52] (Table 2). In addition, the AFB1, CIT, and FB exposure in other plant-based foods including sorghum, peanut, and cowpea may also bring chronic risks of liver cancer, nephrotoxicity, and esophageal cancer [51].

### 2.2. Animal Products

Mycotoxin exposure in animal products arises from the accumulation of mycotoxins in the bodies of animals that consume mycotoxin-contaminated pasture or feed. Although animal products are rich sources of protein and vitamins, their mycotoxin contamination and excessive intake may also increase the risk of exposure to AFs and OTA in the population (Table 2). Data from the Arab countries showed that exposed populations, especially children, are at high risk of exposure to AFs and OTA (0.05–0.98 μg/kg/day) in fried poultry eggs, bovine liver, and kidneys [3,53]. Moreover, a study from Hisar City, Haryana, India, demonstrated that AFM1 contamination can be a food safety issue for raw and pasteurized milk [2]. Of note, the intake of AFB1 in some animal-derived medicines, such as ground beetles and earthworms, poses a slight threat to the cancer risk of the Chinese population [54]. In general, there is less concern about exposure to meats and eggs. Although milk and milk products are of more concern, none of these poses as much concern as the consumption of contaminated cereals.

### 2.3. Fruit and Vegetables and Related Products

Mycotoxins posing health risks in fruit and related products were mainly OTA, HT-2, and AFs. It has been reported that the daily intake of juice has cardiovascular disease prevention benefits; however, it may also lead to exposure to mycotoxins. Data from European countries demonstrated that considerable percentages of OTA and HT-2 TDIs were reached by Spanish children when 200 mL was considered the daily apple fruit juice intake [43]. In addition, data from Türkiye showed that 18.18–57.14% and 71.43–100% of dried fruit samples (such as dried apricots, dried plums, dried figs, and dried grapes) exceeded the European Commission (EC) limits for total AFs and OTA, respectively. Dried fruit intake was associated with a cancer risk for both mycotoxins. Specifically, OTA exposure (with an MOE < 10,000) was determined to pose a risk to public health [55] (Table 2). Studies in recent years have reported that mycotoxin exposure in vegetables posed no health risk to humans.

### 2.4. Spices and Medical Foods

Regarding spice, excessive intake of AFs and OTA is a matter of concern in Asian and African countries. ‘Kankankan’, a popular spice powder in Côte d’Ivoire, is reported to be contaminated with AFs. The EDI of AFs from this spice exceeds the recommended level [38]. A study from Korea emphasized the health concerns related to the genotoxic and carcinogenic potential of AFs in the average intake of homemade soybean paste [56]. Data from Lebanon indicated that AFB1 and OTA levels in some spices, like thyme- and thyme-based products, exceeded the limits. Notably, AFB1 was associated with regular consumption, which was concerning [57,59]. Moreover, the maximum average dietary intake of AFs by individual females in Pakistan was up to 4.80 µg/day/kg in ginger during the winter season, posing great health risks to the local population [58]. A study from Lebanon showed that AFB1 exposure from garlic and onion was also a concern [57] (Table 3).

Regarding medical foods, studies on excessive mycotoxin exposure were mainly from Asian countries (Table 3). Data from China indicated that exposure to AFs in some starch-rich medical foods, like Coix seeds and lotus seeds, may pose risks to human health [42,62]. The latest probabilistic risk assessments for mycotoxins in Coix seeds indicated that AFB1, OTA, and ZEN posed long-term health risks [61]. In addition, a study from Thailand demonstrated a potential risk to consumer health through the consumption of black and white sesame seeds and subsequent exposure to AFB1 [60] (Table 3).

Of note, tea is also one of the main products contaminated with AFs; however, AF exposure from traditional tea in many areas poses no health risks [41]. Only Latvia reported that infants and toddlers were at risk from consuming tea, especially tea made from dog rose fruits [73].

### 2.5. Infant Foods

It is generally believed that food for infants and young children is safer than ordinary food. However, infants and young children have simple dietary sources and low body weights. Levels of estimated dietary exposure to toxins suggest that their health risks are no less than those of the general population. Infants consume large amounts of breast milk. Although breast milk provides macronutrients, infants may be exposed to various mycotoxins during breastfeeding [74,75]. Studies reported that infants in Africa and some areas of the USA were highly exposed to AFM1 through human milk intake [27,65] (Table 4).

Furthermore, infants may be exposed to mycotoxins via contaminated complementary foods, which is an additional concern [65]. Exposure to mycotoxins posing health risks in cereal-based complementary foods mainly included DON, AFs, FBs, OTA, and HT-2. Data from Nigeria showed that AFs contaminated 42% of the cereal- and nut-based foods consumed by infants and young children. The mean concentrations exceeded the EU limits of 0.1 μg/kg set for processed baby foods by 300 times with high chronic exposure and health risks according to the low MOE [64]. Also, in an African country, Tanzania, a study reported that the estimated mean exposure of infants to AFs from maize-based complementary food was 6-fold higher than the health-based guidance value. The proportion of the population at risk of AF exposure above the health-concern limits was 100% [63]. A study from Chile also reported that in almost all the scenarios evaluated, the MOE for AFs in breakfast cereals was less than 10,000, suggesting significant health risks [66]. In addition, data from Nigeria showed that at higher exposure levels, the TWI of 0.1 μg OTA/kg·bw was exceeded by up to 20-fold [64]. A study from Chile reported that the maximum levels of OTA in breakfast cereals led to exposures higher than the TDI [66], which indicated health risks.

Exposure to DON and FBs through infant food intake was also a concern. A study from Tanzania reported that the estimated mean exposures of infants to FBs and DON from maize-based complementary food were 3-fold and 2-fold higher than the health-based guidance values, respectively. The proportion of the population at risk of exposure above the health-concern limits was 35% for DON and 12% for HT-2 toxin [63]. A study from Chile reported that the maximum levels of DON and FBs in breakfast cereals led to exposures higher than the TDI, which indicated some health risks [66]. In addition, some new emerging mycotoxins like ATs were reported to pose health risks to infants by cereal-based food intake. A study from China found that DON, TeA, AOH, and AME were detected in 56.4%, 47.5%, 7.5%, and 5.7% of the cereal-based food samples for Chinese infants and young children. Moreover, 0.2% and 1.5% of individuals exceeded the corresponding reference values for DON and TeA, while 24.1% and 33.5% exceeded the reference values for AME and AOH, respectively [67]. ENNs and BEA in infant foods are also a considerable problem, although the lack of human safety thresholds has limited research to some extent [76] (Table 4).

In addition to calculating the EDI of mycotoxins based on certain food categories, the total dietary study approach was used to calculate the dietary intake of toxins. Reports from Vietnam, using a total dietary study approach, found a significant negative relationship between dietary exposure to individual mycotoxin or mixture and the growth in children and cancer risk in adults [40,77]. It is worth noting that although the risk assessment of finished food products reflects the dietary exposure of the food group from pre-harvest to post-harvest and consumption, inappropriate storage conditions can lead to additional toxin accumulation, thus causing the exposure risk to be underestimated. For example, wheat flour and maize flour may become moldy under suitable temperature and humidity conditions, producing mycotoxins [69,70]. Fruits and vegetables can be further contaminated by *Alternaria* spp. even when stored at low temperatures [78]. Additionally, some non-toxic or low-toxicity hidden toxins may release prototypical mycotoxins during cooking [79]. Therefore, exposure assessments of food raw materials may underestimate the mycotoxin exposure risks when they are processed to an edible state.

## 3. Risk Assessment of Mycotoxin Exposure by Human Biomonitoring Study

**Figure 2:**
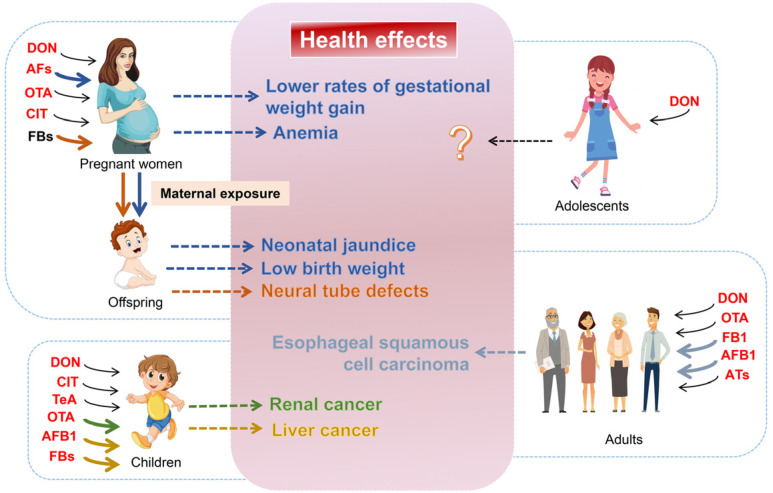
Exposure and health effects of mycotoxins in different populations. The mycotoxin name letters in red indicate toxins whose exposure levels exceed the safety threshold. Bold colored arrows indicate exposure scenarios that have been reported to cause negative health effects; dotted arrows indicate that exposure to toxins with arrows of the same color is associated with the occurrence of this health effect.

Human biomonitoring (HBM) is an efficient approach for intuitively reflecting human exposure to mycotoxins. The biological samples used in HBM methods mainly include blood serum, urine, and breast milk (for infants). To date, mycotoxins have been quantified in many types of human biofluids, including blood, breast milk, stool, and urine. However, exposure assessment data are limited. Demographically stratified HBM data now cover adults, children, adolescents, pregnant women, nursing mothers, and infants. In this review, we summarize the recent progress of the mycotoxin-exposure-profile-based HBM approach, health risks, and health effects (Figure 2).

### 3.1. Risk Assessment of Mycotoxin Exposure in Pregnant Women and Infants

Pregnant women are the key population in public health services, and there are always safety risks during the entire pregnancy process. Notably, many studies have shown that mycotoxin exposure is the key factor causing adverse health effects and pregnancy outcomes in pregnant women [80]. Therefore, mycotoxin exposure and risk assessment are of innovative value and importance for optimizing the experience of pregnancy and health status in pregnant women and improving the quality of the newborn population.

Mycotoxin exposure among pregnant women is widespread worldwide. Studies from rural Ethiopia reported that local pregnant women were co-exposed to at least five mycotoxins. Specifically, one woman was co-exposed to 27 mycotoxins [81]. Studies from rural Bangladesh showed that exposure to multiple mycotoxins during early pregnancy was prevalent in this rural community. Only 17 out of 447 urine samples (4%) from pregnant women had no detectable mycotoxins. Among the detected mycotoxins, dietary exposure to OTA, CIT, and DON was of public health concern. The urine levels of these mycotoxins were associated with the consumption of specific foods and local stimulants such as nuts, betel leaf, and chewing tobacco [82]. Data from China showed that the estimated exposure to DON was 0.96–1.91 μg/kg·bw/day, and approximately 26–60% of individuals in Wuhan exceeded the PMTDI [83].

What is more, studies have shown that mycotoxin exposure may be associated with a negative health status of pregnant women and their fetuses. A study from Uganda found that pregnant women with higher levels of exposure to AFs had lower rates of gestational weight gain, and the association was stronger in HIV-infected women on antiretroviral therapy [84]. Lei et al. demonstrated a correlation between AFB1 exposure during early pregnancy and the risk of anemia, particularly microcytic hypochromic anemia. This correlation varied across different pregnancy trimesters [85]. More importantly, studies have reported that maternal AF exposure during pregnancy can increase the possibility of neonatal jaundice. It may also increase the risk of low birth weight in infants, especially female neonates. This is attributed to the direct and indirect toxicity of AFs, which can trigger maternal systemic inflammation, impair placental growth, and/or elevate placental cytokines [86,87,88,89].

In addition, exposure to FBs among maize-consuming pregnant women has been correlated with neural tube defects in their offspring [90]. A study in Egypt used the benchmark dose approach for genotoxic carcinogens to assess the maternal–fetal risk of OTA during pregnancy. The results indicated that dietary OTA exposure posed a low health risk to this general subpopulation [91].

Although these limited conclusions require more evidence, they are sufficient to warn of the health threat of mycotoxins to these vulnerable groups. Accordingly, the health risk assessment of mycotoxin exposure for pregnant women, nursing mothers, and newborns should be a concern.

### 3.2. Risk Assessment of Mycotoxin Exposure in Children and Adolescents

As children have a higher gastric emptying rate than adults, the health risks caused by exposure to contaminants may be greater for this population. Available reports have validated that children and adolescents face a higher health risk from mycotoxin exposure compared to adults. Data from Norway have shown that children have higher urinary DON concentrations than adults and the elderly [92]. A study from Italy reported that urine samples from children and adolescents had the highest total DON levels, reaching 17.0 ng/mg_creat_. Among children, 40% of individuals exceeded the TDI [93]. An analysis of DON exposure in the Portuguese population revealed that children (3.2%) and adolescents (6.0%) were more likely than adults to exceed the TDI for DON [94]. In both the Henan and Sichuan provinces of China, urinary DON levels varied significantly among different age groups. The levels were highest in adolescents aged 13–17 years, followed by children aged 7–12 years, with a large proportion of individuals exceeding the safety threshold [95].

Data from Germany demonstrated that biomarker concentrations of CIT were higher in children’s urine than in adults’, and 6.3% of individuals exceeded the limit value [44]. Moreover, an assessment of mycotoxin exposure in Spanish children indicated that OTA posed health risks to children [96]. Children in Vietnam were exposed to 52.6 ng/kg·bw/day of OTA. The age-adjusted MOE of renal cancer associated with OTA was 127 (<10,000), indicating potential cancer-related health risks [40]. Data from Vietnam showed that local children were exposed to 118.7 ng/kg·bw/day of AFB1 and 1250.0 ng/kg·bw/day of total FBs, resulting in liver cancer risks of 12.1 cases per 100,000 individuals per year for AFB1 exposure and 542 cases per 100,000 individuals per year for FB exposure, respectively [40].

In the German population, the newly emerging mycotoxin TeA was quantifiable in 97.9% of all samples, ranging from 0.16 to 44.4 ng/mL (average = 6.58 ng/mL). One sample slightly exceeded the TTC of 1.500 μg/kg·bw/day assumed for TeA by the EFSA, indicating a low health risk to humans [97].

### 3.3. Risk Assessment of Mycotoxin Exposure in Adults

For adults, a study in Italy reported that the mean concentration of total DON in urine was 5.54 ng/mg_creat_ for females and 6.84 ng/mg_creat_ for males. The mean EDI of DON was 0.299 μg/kg·bw/day for females and 0.411 μg/kg·bw/day for males, and 7.0% of the adult population exceeded the TDI set for DON [93]. Applying an appropriate model of the association between DON urinary biomarkers and food items, researchers estimated that the median exposure to DON for the Portuguese population was 0.372 µg/kg·bw/day, and 0.1% of the population might exceed the TDI defined for DON [94]. In addition, total DON was detected in 100% and 92% of the urine samples from Henan and Sichuan provinces in China, respectively. DON levels in the urine of Henan subjects (mean: 1.82 μg/kg·bw/day) were significantly higher than those in Sichuan subjects (mean: 0.45 μg/kg·bw/day). Notably, 56% of Henan subjects and 12% of Sichuan subjects were estimated to exceed the PMTDI [95].

Data from Brazil showed that the mean PDIs for OTA and AFB1 were 0.031 and 0.001 μg/kg·bw/day, respectively. Based on urinary biomarkers, the HQ for OTA was greater than 1. For AFs, the MOE values calculated from urine data were below 10,000. These results indicated potential health risks [98]. A descriptive study from Mexico found that the detection rate of AFB1 in serum was up to 91.9%. In terms of population composition, AFB1-lys levels were higher in males (median: 0.195 pg/μL) and older individuals (median: 0.194 pg/μL). Geographically, these levels were higher in rural areas (median: 0.317 pg/μL) compared to urban areas (median: 0.123 pg/μL). Moreover, in populations with a lower socioeconomic status level (median: 0.274 pg/μL), AFB1-lys levels were elevated. These findings suggest that AFs may significantly contribute to the HCC burden among the local population [99]. Data from China also verified that people were exposed to mycotoxins, with concentrations differing between districts. In Jiangsu Province, AFB1 was detected in paired plasma and first-morning urine samples and indicated a potential public health concern with a mean daily intake of 0.41 μg/kg·bw/day [100]. Xue et al. found that AFB1 and FB1 exposure levels were significantly higher in esophageal squamous cell carcinoma cases (median: 1.77 pg/mg albumin for AFB1 and 176.13 pg/mg_creat_ for FB1) than in controls (median: 1.49 pg/mg albumin for AFB1 and 56.92 pg/mg_creat_ for FB1), which was significantly associated with an increased risk of ESCC in the local area [101].

Regarding “emerging” mycotoxins, Fan et al. detected AOH, AME, TeA, and TEN in 38.3%, 48.7%, 63.9%, and 23.4% of urine samples, respectively. The median PDIs for these mycotoxins were 19.5 ng/kg·bw/day for AOH, 12.3 ng/kg·bw/day for AME, 1.75 ng/kg·bw/day for TeA, and 27.8 ng/kg·bw/day for TEN. Moreover, 100% of participants exceeded the TTC values for AOH and AME, while 0.372% and 1.12% exceeded the TTC values for TeA and TEN [37], revealing the potential health risks caused by the contamination of major ATs in China. Despite the lack of health guidance values for ENNs and BEA, the EFSA Panel on Contaminants in the Food Chain noted potential risks associated with chronic exposure to BEA and ENNs [102].

## 4. Discussion

Mycotoxin contamination is an urgent global problem. Since the toxicity of mycotoxins was discovered, researchers have devoted significant effort to the harmful control of mycotoxins, such as removal, detoxification, and degradation by physical, chemical, and biological methods. However, these methods are not widely used due to safety and other issues. Accordingly, studies related to mycotoxin exposure risks to humans have gradually increased with the widespread occurrence of mycotoxins in foods.

Mycotoxin contamination affects numerous food categories. Dietary exposure assessments based on food consumption showed that toxin exposures exceeding safe thresholds occur in cereals, fruits and vegetables, animal products, certain spices, and medicinal foods, with cereals being a major concern (Table 2). In cereals, dietary exposures to DON, FBs, OTA, CIT, and AFB1 were widespread in different populations, especially children and adolescents. Exposure to dietary toxins has been reported in both developed and developing countries. However, there are geographical specificities. For example, excessive toxin intake from spices and medicinal diets has been reported in the Asian region, while in European countries, it is mainly from fruits and related products, and in Arab countries, it is mainly from animal products.

**Figure 3:**
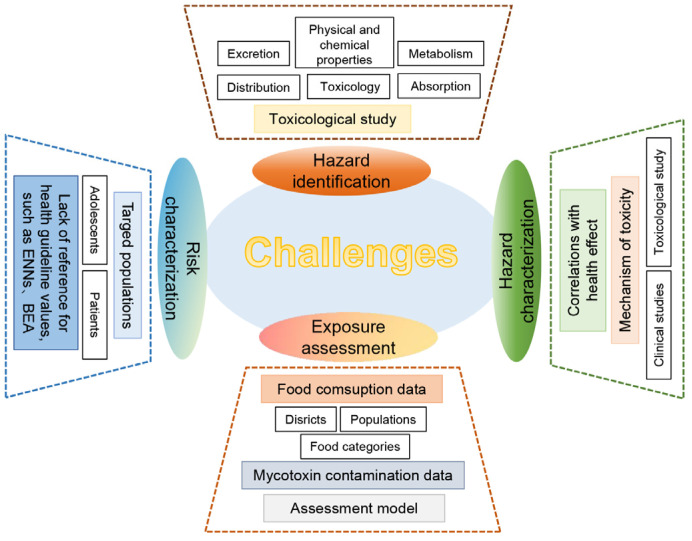
Challenges in mycotoxin exposure assessments.

Risk assessment of mycotoxins using HBM of biological samples like serum and urine provides a more intuitive understanding of the extent of human mycotoxin exposure. In the studies of recent years, mycotoxins have been prevalent in all types of biological samples and pose varying degrees of health risk in different populations, with more data from developed countries regarding child and adult populations. In particular, it can be found that infants and young children are at a higher risk of mycotoxin exposure. However, for the HBM of this population, data are scarcer, which should be a concern for future research. Meanwhile, these studies highlight the necessity for more in vivo toxicological studies to provide a better basis for further comprehensive risk assessments, especially for some emerging mycotoxins. Despite growing research on mycotoxin exposure risks, there are still many gaps and challenges in current related research (Figure 3). Regarding hazard identification, for some emerging toxins, there is still a lack of comprehensive technical information analysis support from authorities. Toxicological studies covering aspects such as basic toxicology, physical and chemical properties, absorption and metabolism regulation, toxicokinetics, and tissue distribution are required. These studies should be carried out in line with the guidelines recommended by international organizations or relevant national standards. For hazard characterization, specific toxicity and health hazards to humans of toxins need to be illustrated by toxicological and clinical studies. For exposure assessment, there is currently a lack of data on a significant portion of food consumption of key populations such as pregnant women, infants, and specific patient populations, which needs to be further explored.

More importantly, effective risk assessment models need to be developed. A study published in 2024 proposed a tiered hazard-prioritization and risk-ranking approach, which included two steps: exposure-based screening and MOE-based probabilistic risk ranking [76]. This may become a promising tool for quantifying and prioritizing health risks in support of human health risk management. In addition, researchers evaluated the potential risk of chronic exposure to ENNs in the worst-case scenario using wastewater-based epidemiology [103], which provides a novel perspective for assessing mycotoxin exposure risks. In addition, integrating artificial intelligence, big data, and the Internet of Things into food safety early-warning and emerging-risk-identification tools may hold promise for better managing mycotoxin exposure risks. Regarding the risk characterization of mycotoxins, the lack of health guideline values for mycotoxins, especially emerging mycotoxins, can introduce uncertainties (such as the TTC for ATs). Moreover, it can limit risk assessment studies (for example, due to the absence of health guideline reference values for ENNs and BEA). In addition, hazard characterization should be qualitatively or quantitatively described in terms of the relationship between hazards and different health effects (toxic endpoints) and mechanisms of action.

## Figures and Tables

**Table 1:** Health-based guidance values for mycotoxins.

Mycotoxin	TDI or PMTDI (µg/kg·bw/day)	References
NIV	1.2	[39]
FBs	2.0	
OTA	0.017	
DON	1.0	
T-2 and HT-2	0.10	
HT-2	0.060	[42]
ZEN	0.25	[39]
PAT	0.40	[43]
CIT	0.20	[44]
TeA	1.5 ^1^	[37]
TEN	1.5 ^1^	
AOH	0.0025 ^1^	
AME	0.0025 ^1^	

^1^ indicated TTC value.

**Table 2:** Distribution of toxins with health risks (EDI > TDI or MOE < 10,000) in different food categories.

Food Category	Sample Type	Mycotoxin	Time	EDI (µg/kg·bw/day)			Country
				Adults	Teenagers	Children	
Cereal	Rice, maize, wheat, and their products	FBs	2020 [45]	10–114 years old: 99th percentile (P99) 2.440 Teenagers: P97.5 2.110 Women of child-bearing age: P99 2.090			Brazil (South America)
		DON		10–114 years old: P99 1.260 Teenagers: P95 1.040			
	Wheat flour	DON	2016 [46]	–	1.200	–	
	Wheat	DON	2017–2019 [47]	0.000–2.730	–	–	China (Asian)
	Wheat grains	AOH	2021 [48]	4–70 years old: 0.004–0.007			
		AME		4–70 years old: 0.004–0.007			
	Beer	OTA	2018 [49]	0.007–0.023	–	–	Portugal (Europe)
	Maize	DON	2020 [50]	Men: P95 1.280	–	–	Mexico (North America)
		FBs	2018–2019 [51]	3.240	7.880	19.700	Nigeria (Africa)
		FB1 + FB2	2020 [50]	2.340	–	–	Mexico
		AFB1	2018–2019 [51]	0.108	0.262	0.655	Nigeria
		CIT		2.540	6.180	15.400	
	Rice	OTA	2018 [52]	–	–	P99 0.002	Belgium (Europe)
		CIT	2018 [52]	–	–	0.006	
			2018–2019 [51]	0.080	0.210	0.530	Nigeria
		AFB1	2018–2019 [51]	0.032	0.078	0.194	
Other plant-based foods	Peanut	AFB1	2018–2019 [51]	0.803	1.953	4.883	Nigeria
		CIT		0.090	0.220	0.550	
Animal products	Poultry eggs	AFB1	2017 [3]	9.490 × 10^−5^–1.010 × 10^−4^	–	6.020 × 10^−4^–2.090 × 10^−4^	Jordan (Arab)
	Milk	AFs	2018 [2]	0.001–0.005	–	–	India (Asian)
	Bovine meat and edible offal		2019 [53]	0.050–0.420	–	0.110–0.980	Al-Ahsa (Arab)
	Medicines	AFB1	2021 [54]	0.274 × 10^−3^	–	–	China
Fruits	Apple juice	HT-2	2018 [43]	–	–	0.180	Spanish (Europe)
		OTA	2018 [43]	–	–	0.04	
	Dried fruits		2021 [55]	11 × 10^−6^–60 × 10^−6^	–	–	Türkiye (Europe)

**Table 3:** Distribution of toxins with health risks (EDI > TDI or MOE < 10,000) in spices and medical foods.

Food Category	Sample Type	Mycotoxin	Time	EDI (µg/kg·bw/day)	Country
				Adults	
Spices	Kankankan	AFB1	2017 [38]	0.007–0.035	Côte d’Ivoire (Africa)
	Homemade soybean pastes	AFs	2018–2020 [56]	0.018 × 10^−3^–0.548 × 10^−3^	Korea (Asian)
	Spices for Lebanese dishes	AFB1	2020 [57]	0.001–0.004	Lebanon (Asian)
	Ginger	AFs	2022 [58]	0.001–0.004	Pakistan (Asian)
	Thyme and thyme-based products	AFB1	2022 [59]	0.004–0.005	Lebanon (Asian)
		OTA		0.001	
Medical foods	Sesame seeds	AFs	2020 [60]	0.040 × 10^−3^	China (Asian)
	Coix seeds, malt, lotus seeds, and lilii bulbus	AFB1	2020–2022 [42]	Male 0.008 Female 0.010	
		HT-2		Female 0.068	
	Coix seeds	AFs	2023 [61,62]	0.003–0.014	
		ZEN		P97.5 0.272	

**Table 4:** Distribution of toxins with health risks (EDI > TDI or MOE < 10,000) in infant foods.

Sample Type	Mycotoxin	Time	EDI (µg/kg·bw/day)	Country
Maize-based complementary foods	AFs	2016 [63]	0.133	Tanzania (Africa)
	FBs		12.000	
	DON		1.900	
Complementary foods	AFs	2017 [64]	0.026–54.892	Nigeria (Africa)
	CIT		0.002–102.000	
	BEA		0.000–3.140	
Breast milk	AFB1	2018 [65]	1.400 × 10^−3^–16.100 × 10^−3^	Ecuadorian highlands (South America)
	HT-2		0.062–0.529	
	OTA		0.003–0.032	
Breakfast cereal	AFs	2019–2021 [66]	0.001–0.006	Chile (South America)
	FBs		0.019–3.262	
	DON		0.036–2.512	
	OTA		0.300 × 10^−3^–4.000 × 10^−3^	
Cereal-based food products	DON	2021 [67]	Maximum 6.480	China (Asian)
	AME		P90 0.004	
	AOH		0.003	
	TeA		Maximum 3.500	

## Data Availability

No new data were created or analyzed in this study. Data sharing is not applicable to this article.

## Abbreviations

The following abbreviations are used in this manuscript:

DON	deoxynivalenol
AFs	aflatoxins
OTs	ochratoxins
ZEN	zearalenone
FBs	fumonisins
PAT	patulin
CIT	citrinin
ATs	*Alternaria* toxins
AOH	alternariol
AME	alternariol monomethyl ether
TeA	tenuazonic acid
TEN	tentoxin
ALT	altenuene
ENNs	enniatins
BEA	beauvericin
IARC	International Agency for Research on Cancer
EU	European Union
EDI	estimated daily intake
PDI	probable daily intake
PMTDI	provisional maximum tolerable daily intake
TDI	tolerable daily intake
TWI	tolerable weekly intake
FAO/WHO	Food and Agriculture Organization of the United Nations/World Health Organization
JECFA	Joint FAO/WHO Expert Committee on Food Additives
EFSA	European Food Safety Authority
TTC	threshold of toxicological concern
HQ	hazard quotient
MOE	margin of exposure
ADI	acceptable daily intake
EWI	estimated weekly intake
EC	European Commission
HBM	human biomonitoring
